# Draft genome sequence of *Dethiosulfovibrio salsuginis* DSM 21565^T^ an anaerobic, slightly halophilic bacterium isolated from a Colombian saline spring

**DOI:** 10.1186/s40793-017-0303-x

**Published:** 2017-12-20

**Authors:** Carolina Díaz-Cárdenas, Gina López, José David Alzate-Ocampo, Laura N. González, Nicole Shapiro, Tanja Woyke, Nikos C. Kyrpides, Silvia Restrepo, Sandra Baena

**Affiliations:** 1 Unidad de Saneamiento y Biotecnología Ambiental, Departamento de Biología, Pontificia Universidad Javeriana, POB 56710, Bogotá, DC Colombia; 2 Biological Sciences Department, Universidad de los Andes, Cra 1 No. 18A-12, Bogotá, DC Colombia; 3 Department of Energy, Joint Genome Institute, Walnut Creek, CA 94598 USA

**Keywords:** *Dethiosulfovibrio salsuginis*, *Synergistetes*, Halophilic, Anaerobe, Fermentation of amino acids, Saline spring

## Abstract

A bacterium belonging to the phylum *Synergistetes*, genus *Dethiosulfovibrio* was isolated in 2007 from a saline spring in Colombia. *Dethiosulfovibrio salsuginis* USBA 82^T^ (**DSM 21565** ^**T**^ **= KCTC 5659** ^**T**^ **)** is a mesophilic, strictly anaerobic, slightly halophilic, Gram negative bacterium with a diderm cell envelope. The strain ferments peptides, amino acids and a few organic acids. Here we present the description of the complete genome sequencing and annotation of the type species *Dethiosulfovibrio salsuginis* USBA 82^T^. The genome consisted of 2.68 Mbp with a 53.7% G + C*.* A total of 2609 genes were predicted and of those, 2543 were protein coding genes and 66 were RNA genes. We detected in USBA 82^T^ genome six *Synergistetes* conserved signature indels (CSIs), specific for *Jonquetella, Pyramidobacter* and *Dethiosulfovibrio*. The genome of *D. salsuginis* contained, as expected, genes related to amino acid transport, amino acid metabolism and thiosulfate reduction. These genes represent the major gene groups of *Synergistetes*, related with their phenotypic traits, and interestingly, 11.8% of the genes in the genome belonged to the amino acid fermentation COG category. In addition, we identified in the genome some ammonification genes such as nitrate reductase genes. The presence of proline operon genes could be related to de novo synthesis of proline to protect the cell in response to high osmolarity. Our bioinformatics workflow included antiSMASH and BAGEL3 which allowed us to identify bacteriocins genes in the genome.

**Electronic supplementary material:**

The online version of this article (doi: 10.1186/s40793-017-0303-x) contains supplementary material, which is available to authorized users.

## Introduction

The bacteria belonging to the phylum 10.1601/nm.14317, a robust monophyletic branch of the phylogenetic tree based on rRNA data, are widespread in a wide range of anoxic ecosystems. Jumas-Bilak & Marchandin [1] have delineated several habitats in which the members of this phylum live. These include sludge and wastewater from anaerobic digesters [2–4], natural springs [5], natural seawater and sulfur mats [6], water related to petroleum and gas production facilities [7, 8] and host-associated microbiota [9–11].

A distinguishing feature which is common to all members of the phylum 10.1601/nm.14317 [12] is the capacity to use amino acids as sources of energy [13]. The ability to ferment carbohydrates is limited to a few cultured species [4]. Currently, the phylum groups 15 genera: 10.1601/nm.11328 *,* 10.1601/nm.24591 *,* 10.1601/nm.4469 *,* 10.1601/nm.4472 *,* 10.1601/nm.13071 *,* 10.1601/nm.10182 *,* 10.1601/nm.23836 *,* 10.1601/nm.11530 *,* 10.1601/nm.14314 *,* 10.1601/nm.620 *,* 10.1601/nm.4502 *,* 10.1601/nm.25564 *,* 10.1601/nm.4487 *,* 10.1601/nm.7999 and 10.1601/nm.27929 [4, 14–23]. They include 28 species of strictly anaerobic, neutrophilic, Gram-negative bacteria. The genus 10.1601/nm.4487 comprises five described species: 10.1601/nm.4488 [7], the type species of the genus; 10.1601/nm.4489 *,* 10.1601/nm.4490 *,* 10.1601/nm.4491 [6] and 10.1601/nm.17912 [5] which were isolated from corroding offshore oil wells, ‘Thiodendron’ sulfur mats in various saline environments and a Colombian saline spring. Members of the genus 10.1601/nm.4487 are vibrios or curved or vibrioid-like rods which are mesophilic, neutrophilic, slightly halophilic, chemoorganoheterotrophic, sulfur and thiosulfate-reducing bacteria. They share 98.5% of their 16S rRNA gene sequence positions with the type species of the genus, 10.1601/nm.4488 *,* and only 94.2% with the fifth characterized species of the genus, 10.1601/nm.17912 [1].

Bhandari and Gupta [24] identified molecular markers consisting of conserved signature insertions/deletions (indels) (CSIs) present in protein sequences which are specific for 10.1601/nm.14317 *.* Of these, seven are specifically present in 10.1601/nm.11530 *,* 10.1601/nm.14314 and 10.1601/nm.4487. In this study, we verified whether these CSIs are also present in 10.1601/nm.17912 USBA 82^T^.

## Organism information

### Classification and features

10.1601/nm.17912 USBA 82^T^ was isolated in 2007 from the saline spring named Salpa, in the Colombian Andes. The spring has a temperature ~ 21 °C and pH ~ 6.5 throughout the year. The predominant dissolved ion is sulfate (20 g.l^−1^) and the conductivity is approximately 50 mS. cm^−1^ [25]. Samples were collected in sterile containers, which were capped, stored over ice, transported to the laboratory and maintained at 4 °C until use [5]. Enrichments were done as described in Díaz-Cárdenas et al. [5]. Briefly, they were initiated in a medium prepared by filtering saline spring water through polycarbonate membranes (Durapore) with a pore size of 0.22 μm. The medium was supplemented with peptone (0.2%, *w*/*v*), yeast extract (0.02%, w/v) and the trace element solution (1 ml l^−1^) as described by Imhoff-Stuckle & Pfenning [26]. Then, the medium was boiled and then cooled to room temperature under a stream of oxygen-free nitrogen. An 8 ml aliquot was dispensed into Hungate tubes under oxygen-free nitrogen gas and sterilized by autoclaving at 121 °C for 20 min at a pressure of 1–1.5 kg cm^−2^. The enrichment medium was inoculated with 2 ml water samples, incubated at 36 °C for up to 2 weeks. To isolate pure cultures, serial dilutions of the enrichment cultures were made in an artificial basal medium (BM) fortified with 2% (w/v) Noble agar (pH = 7.1) using the roll-tube technique [5].

**Fig. 1:**
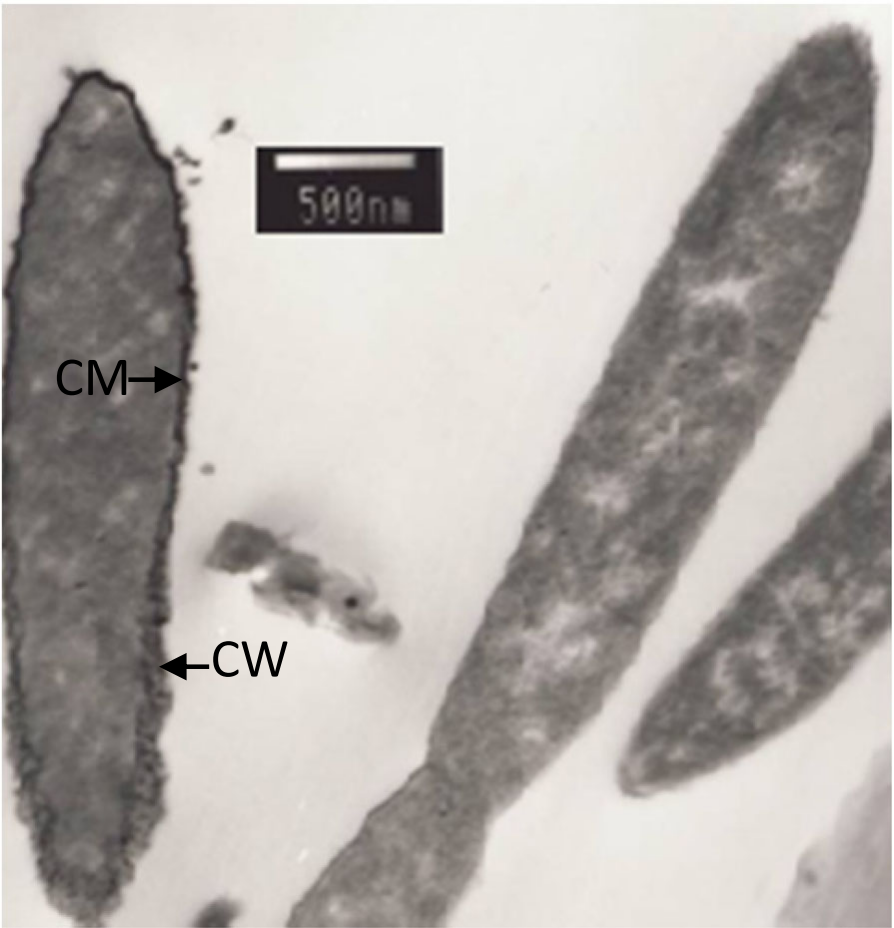
Electron micrograph of negatively stained cells of strain USBA 82^T^. An ultra-thin section revealing the presence of a typical Gram-negative cell wall ultra-structure CW: cell wall; CM, cytoplasmic membrane (Bar = 500 nm)

Cells of strain USBA 82^T^ are slightly curved rods with pointed or rounded ends (5–7 × 1.5 μm) and occur singly or in pairs. Cells are motile by laterally inserted flagella (Fig. 1). This non-spore-forming, strictly anaerobic, slightly halophilic, Gram negative bacterium with a diderm cell envelope, presents some particular metabolic features. It ferments arginine, casamino acids, glutamate, histidine, peptone, serine, threonine, tryptone, pyruvate and citrate, but growth is not observed on carbohydrates, alcohols or fatty acids. The main end products of fermentation are acetate and succinate [5]. As other members of the genus, strain USBA 82^T^ reduces thiosulfate and sulfur to sulfide but sulfate, sulfite, nitrate and nitrite are not used as electron acceptors [5]. The reduction of sulfur or thiosulfate is not required for growing on the amino acids arginine, glutamate and valine. The strain USBA 82^T^ ferments these amino acids, in contrast to that observed on 10.1601/nm.4488 *.*

The strain USBA 82^T^ grows optimally at 30 °C (growth range 20–40 °C), pH 7.3 (pH growth range pH 5.5–8.5) and 2% (*w*/*v*) NaCl (growth range 0.1–7% NaCl) [5].

The isolate was assigned to the phylum 10.1601/nm.14317, close to 10.1601/nm.4488, by comparison of the 16S rRNA sequence with a similarity value of 94.2% [5, 7]. Comparison of the phylogenetic, chemotaxonomic and physiological features of strain USBA 82^T^ with all other members of 10.1601/nm.4487, suggested that it represents a novel species for which the name 10.1601/nm.17912 was proposed [5].

10.1601/nm.17912 was stored since the collection date at the Collection of Microorganisms of Pontificia Universidad Javeriana (CMPUJ, WDCM857) (ID CMPUJ U82^T^ =10.1601/strainfinder?urlappend=%3Fid%3DDSM+21565 ^T^=10.1601/strainfinder?urlappend=%3Fid%3DKCTC+5659 ^T^) with the ID USBA 82^T^ growing anaerobically on the BM medium described by Díaz-Cárdenas et al. [5]. Cells are preserved at −20 °C in BM supplemented with 20% (*v*/v) glycerol [5]. The general features of the strain are reported in Table 1.

**Table 1:** Classification and general features of *D. salsuginis* according to MIGS standards [28]

MIGS ID	Property	Term	Evidence code^a^
	Current classification	Domain: *Bacteria*	TAS [57]
		Phylum: *Synergistetes*	TAS [12]
		Class: *Synergistia*	TAS [12]
		Order: *Synergistales*	TAS [12]
		Family: *Synergistaceae*	TAS [12]
		Genus: *Dethisulfovibrio*	TAS [7]
		Species: *D. salsuginis* Type strain is USBA 82 (DSM 21565^T^= KCTC 5659^T^)	TAS [5]
	Gram-stain	Negative	TAS [5]
	Cell shape	slightly curved rods with pointed or rounded ends	TAS [5]
	Motility	Motile	TAS [5]
	Sporulation	Negative	TAS [5]
	Temperature range	20–40 °C	TAS [5]
	Optimum temperature	30 °C	TAS [5]
	pH range; Optimum	5.5–8.5, 7.3	TAS [5]
	Carbon source	Peptone, casaminoacids and AA	TAS [5]
	Energy source	Chemoheterotrophic	TAS [5]
MIGS 6	Habitat	Saline Spring	TAS [5]
MIGS-6.3	Salinity	2% NaCl (w/v)	TAS [5]
MIGS 22	Oxygen requirement	Strictly anaerobic	TAS [5]
MIGS 15	Biotic relationship	free-living	TAS [5]
MIGS 14	Pathogenicity	unknown	TAS [5]
	Biosafety level	unknow	TAS [5]
MIGS 4	Geographic location	Colombia	TAS [5]
MIGS 5	Sample collection time	2007	TAS [5]
MIGS 4.1	Latitude	05°46′09.2”N	TAS [5]
MIGS 4.2	Longitude	73°06′09.7” W	TAS [5]
MIGS-4.4	Altitude	2517 m.a.s.l.	TAS [5]

^a^Evidence codes: *IDA* inferred from direct assay (first time in publication), *TAS* traceable author statement (i.e., a direct report exists in the literature), *NAS* non-traceable author statement (i.e., not directly observed for the living, isolated sample, but based on a generally accepted property for the species, or anecdotal evidence). These codes are from the Gene Ontology project [58]

## Genome sequencing information

### Genome project history

Jumas-Bilak & Marchandin [1] pointed out that bacteria belonging to the phylum 10.1601/nm.14317 remain poorly characterized by molecular approaches, particularly by typing methods, and the only gene sequences currently available for most organisms of the phylum are 16S rDNA sequences. Currently, there are twenty-eight isolates that are fully sequenced and annotated or in the phase of final sequencing. The type strain USBA 82^T^ was selected to sequencing on the basis of its novelty and this genome contributes with the Genomic Encyclopedia of Bacteria and Archaea [27]. In addition, this work is part of the bigger study aiming at exploring the microbial diversity in extreme environments in Colombia. More information can be found on the Genomes OnLine database under the study Gs0118134. The JGI accession number, sequence project ID is 1,094,809 and consists of 68 scaffolds. The annotated genome is publically available in IMG under Genome ID FXBB01000001-FXBB01000068. Table 2 depicts the project information and its association with MIGS version 2.0 compliance [28].

**Table 2:** Project information

MIGS ID	Property	Term
MIGS 31	Finishing quality	High-quality draft
MIGS 28	Libraries used	Paired-end
MIGS 29	Sequencing platforms	Illumina HiSeq 2500
MIGS 30	Assemblers	ALLPATHS/Velvet
MIGS 32	Gene calling method	BLAST2GO
	Locus Tag	Not indicated
	JGI ID (Seq project)	1,094,809
	JGI Date of Release	January 29, 2016
	GOLD ID	Gs0118134
MIGS 13	Source Material Identifier	USBA 82
	Project relevance	Metabolic versatility, natural products discovery

### Growth conditions and genomic DNA preparation (heading level 2)

10.1601/nm.17912 strain USBA 82^T^ was grown anaerobically on 100 mL of BM supplemented with 1.0 g yeast extract and 0.5% (w/v) peptone [5] at 30 °C for 24 h. The growth was monitored by OD_580nm_. Cells were harvested by centrifugation at 4000 rpm when the mid exponential phase (OD_580nm_ = 0.2) was reached, pelleted and immediately used for DNA extraction. We extracted the genomic DNA using the Wizard® Genomic DNA Purification Kit (Promega) according to the manufacturer’s instructions.

### Genome sequencing and assembly

The draft genome of 10.1601/nm.17912 was generated at the DOE Joint Genome Institute (JGI) using the Illumina technology [29]. An Illumina 300 bp insert standard shotgun library was constructed and sequenced using the Illumina HiSeq 2500 platform which generated 12,750,038 reads totaling 1912.5 Mbp. All general aspects of library construction and sequencing performed at the JGI can be found at http://www.jgi.doe.gov. All raw Illumina sequence data was filtered using BBDuk [30], which removes known Illumina artifacts and PhiX. Reads with more than one “N” or with quality scores (before trimming) averaging less than 8 or reads shorter than 51 bp (after trimming) were discarded.

Remaining reads were mapped to masked versions of human, cat and dog references using BBMAP [30] and discarded if identity exceeded 93%. Sequence masking was performed with BBMask [30]. The following steps were then performed for assembly: (1) artifact filtered Illumina reads were assembled using Velvet (version) [31]; (2) 1–3 kbp simulated paired end reads were created from Velvet contigs using wgsim (version 0.3.0) [32]; (3) Illumina reads were assembled with simulated read pairs using Allpaths–LG (version r46652) [33]. Parameters for assembly steps were: (1) Velvet (velveth: and velvetg), (2) wgsim (−e 0–1100–2100 –r 0 –R 0 –X 0), (3) Allpaths–LG (PrepareAllpathsInputs: PHRED 64 = 0 PLOIDY = 1 FRAG COVERAGE = 125 JUMP COVERAGE = 25 LONG JUMP COV = 50 and RunAllpathsLG: THREADS = 8 RUN = std. shredpairs TARGETS = standard VAPI WARN ONLY = True OVERWRITE = True).

### Genome annotation

Annotation was done using the DOE-JGI annotation pipeline [34]. Genes were identified using Prodigal [35]. The predicted CDSs were translated and used to search the National Center for Biotechnology Information nonredundant database, UniProt, TIGRFam, Pfam, KEGG, COG, KOG, MetaCyc (version 19.5) and Gene Ontology databases. The first category of non-coding RNAs, tRNAs, were predicted using tRNAscan-SE 1.3.1 tool [36] Ribosomal RNA genes (5S, 16S, 23S) were predicted using hmmsearch tool from the package HMMER 3.1b2 [37]. Other non–coding RNAs such as the RNA components of the protein secretion complex and the RNase P were identified by searching the genome for the corresponding Rfam profiles using INFERNAL [38]. Additional gene prediction analysis and manual functional annotation was performed within the Integrated Microbial Genomes – Expert Review platform [39] developed by the Joint Genome Institute, Walnut Creek, CA, USA. The annotated genome of strain USBA 82^T^ is available in IMG (genome ID = 2,671,180,116).

We used IMG tools for data mining to explore potential production of secondary metabolites of 10.1601/nm.17912 genome. In addition, we developed a bioinformatics workflow which included platforms such as antiSMASH [40], BAGEL3 [41] and NaPDoS [42].

## Genome properties

The genome of 10.1601/nm.17912 is 2.68 Mbp with a 53.7% GC content. A total of 2609 genes were predicted and of those, 2543 were protein coding genes and 66 were RNA genes. The properties and statistics of the genome are summarized in Table 3. The distribution of genes into COGs functional categories is presented in Table 4. Most genes were classified in the category of amino acid transport and metabolism (11.8%), followed by general function (8.3%) and inorganic ion transport and metabolism (6.6%).

**Table 3:** Genome statistics

Attribute	Value	% of Total
Genome size (bp)	2,681,495	100
DNA coding (bp)	248,306	92.60
DNA G + C (bp)	1,439,962	53.7
DNA scaffolds	68	100
Total genes	2609	100
Protein coding genes	2543	97.47
RNA genes	66	2.59
Pseudo genes	7	0.26
Genes in internal clusters	516	19.77
Genes with function prediction	2062	79.03
Genes assigned to COGs	1747	66.96
Genes with Pfam domains	2126	81.48
Genes with signal peptides	169	6.47
Genes with transmembrane helices	630	24.14
CRISPR repeats	1

**Table 4:** Number of genes associated with general COG functional categories

Code	Value	% age	Description
J	171	8.81	Translation, ribosomal structure and biogenesis
A	0	0	RNA processing and modification
K	104	5.36	Transcription
L	73	3.76	Replication, recombination and repair
B	0	0	Chromatin structure and dynamics
D	27	1.39	Cell cycle control, Cell división, chromosome partitioning
V	46	2.37	Defense mechanisms
T	135	6.95	Signal transduction mechanisms
M	108	5.56	Cell wall/membrane biogenesis
N	86	4.43	Cell motility
U	28	1.44	Intracellular trafficking and secretion
O	71	3.66	Posttranslational modification, protein turnover, chaperones
C	119	6.13	Energy production and conversion
G	106	5.46	Carbohydrate transport and metabolism
E	229	11.79	Amino acid transport and metabolism
F	68	3.50	Nucleotide transport and metabolism
H	103	5.30	Coenzyme transport and metabolism
I	44	2.27	Lipid transport and metabolism
P	129	6.64	Inorganic ion transport and metabolism
Q	18	0.93	Secondary metabolites biosynthesis, transport and catabolism
R	161	8.29	General function prediction only
S	85	4.38	Function unknown
–	888	33.70	Not in COGs

The total is based on the total number of protein coding genes in the genome; COG was obtained from the JGI IMG pipeline [34]

## Insights from the genome sequence

The draft genome provides phylogenetic and metabolic information. Phylogenetic relationship was evaluated using 16S rRNA gene sequence and seven conserved signature indels identified as specific for a clade consisting of 10.1601/nm.11529, 10.1601/nm.14315 and 10.1601/nm.4488 [24].

**Fig. 2:**
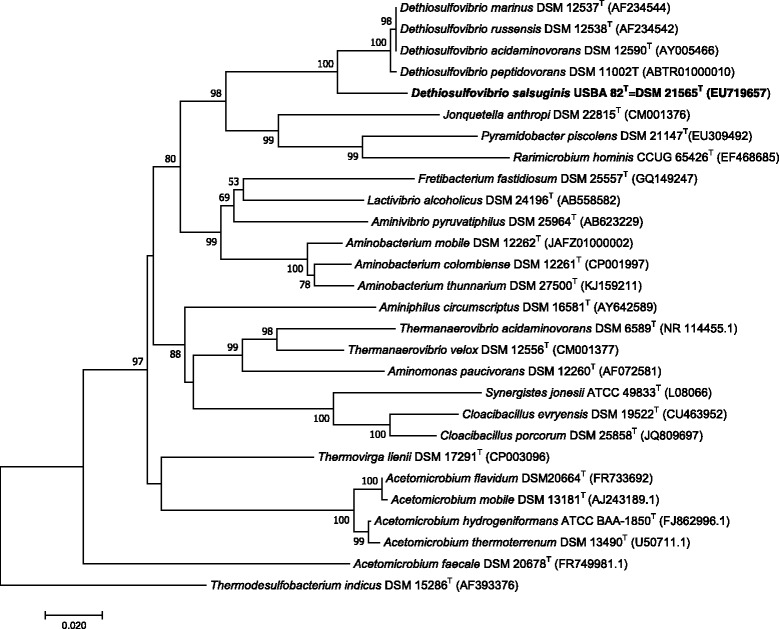
Relationships of *D. salsuginis* USBA 82^T^ using 16S rRNA gene was inferred using the Neighbor-Joining method. The percentage of replicate trees in which the associated taxa clustered together in the bootstrap test (1000 replicates) is shown next to the branches. The evolutionary distances were computed using the Jukes-Cantor method and are in the units of the number of base substitutions per site. The analysis involved 28 nucleotide sequences. All positions containing gaps and missing data were eliminated. There was a total of 1085 positions in the final dataset. Evolutionary analyses were conducted in MEGA7

Sequences of the 16S rRNA gene of strain USBA 82^T^ and related strain types currently characterized in the phylum 10.1601/nm.14317 were aligned using MEGA 7 program version 7.0.25 [43]. The evolutionary distance was analyzed by Neighbour-Joining (NJ) [44], using Jukes-Cantor method [45] (Fig. 2) and Maximum-Likelihood (ML) using the General Time Reversible (GTR) model plus gamma distribution and invariant sites see Additional file 1: Figure S1) [46]. Bootstrap support was computed after 1000 reiterations for NJ and ML analysis. 10.1601/nm.502 10.1601/strainfinder?urlappend=%3Fid%3DDSM+15286 ^T^ (GenBank accession number AF393376) was used as outgroup in all phylogenetic analyses. The topology of the trees confirmed that the strains belong to subdivision B of the phylum 10.1601/nm.14317 together with members of the genera 10.1601/nm.4487 *,* 10.1601/nm.11530 *,* 10.1601/nm.14314 and 10.1601/nm.27929.

We compared seven conserved signature indels that are present in the following protein sequences: penicillin binding protein, 1A family; ribonucleoside diphosphate reductase (*nrdA*); putative DEAD/DEAH box helicase (indel position 398–457); putative DEAD/DEAH box helicase (indel position 437–496); DNA directed RNA polymerase, ß subunit (*rpoB*); 1-acyl-sn-glycerol-3-phosphate acyltransferase (*plsC*) and tRNA modification GTPase TrmE (*trmE*). We used an identification pipeline with BlastP [43] searches of the reported CSIs over the genome of 10.1601/nm.17912 USBA 82^T^ and 10.1601/nm.11529 10.1601/strainfinder?urlappend=%3Fid%3DDSM+22815/1–750, 10.1601/nm.14315 W 5455/1738 and 10.1601/nm.4488 10.1601/strainfinder?urlappend=%3Fid%3DDSM+11002/1–743, and multiple alignments using Mafft [44]. The indels that we detected correspond in size to those previously reported by Bhandari and Gupta [24]. We found a 4 amino acids (aa) deletion in the penicillin binding protein, 1A family (see Additional file 2: Figure S2), a 1aa insertion in the *nrdA* gene (see Additional file 3: Figure S3), a 13aa insertion in the *rpoB* gene (see Additional file 4: Figure S4), a 1aa insertion in the *plsC* gene (see Additional file 5: Figure S5) and a 1aa insertion in the *trmE* gene (see Additional file 6: Figure S6). DEAD/DEAH box CSIs were neither detected in our genome, nor have they ever been detected in previously analyzed species (see Additional file 7: Figure S7).

We also evaluated ultrastructure characters including the cell-wall structure, which currently supports the separation of the 10.1601/nm.14317 clade from other members of the family 10.1601/nm.4462. We detected the presence of a particular deletion in the Hsp60 protein in USBA 82^T^ (see Additional file 8: Figure S8). It differentiates the traditional Gram-negative diderm bacterial phyla from atypical taxa of diderm bacteria such as 10.1601/nm.19297, ‘10.1601/nm.8343 *’*, ‘10.1601/nm.17781 *’* and 10.1601/nm.14317 [47]. It has been reported that 10.1601/nm.14317 species contain an outer membrane and also have genes that are used for lipopolysaccharide biosynthesis in other microorganisms. However, they lack the genes for the TolAQR-Pal complex that are required for assembly and maintenance of typical outer membrane [48] suggesting that the nature and the role of the outer membrane in 10.1601/nm.14317 could be different than those of other bacteria. This observation was also confirmed in the 10.1601/nm.17912 strain USBA 82^T^ genome.

**Fig. 3:**
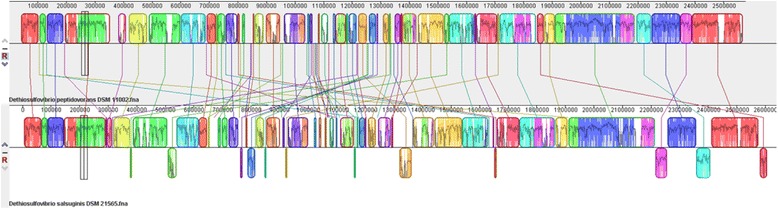
Multiple Alignment performed using Mauve of *D. salsuginis* USBA-82^T^ and *D. peptidovorans* DSM 11002^T^ genomes. The type strain of D. peptidovorans (DSM 11002^T^) is shown at the botton and the strain USBA-82^T^ (DSMZ 21565^T^) is shown in the top. Conserved blocks are represented with direct lines from *D. peptidovorans* to strain USBA-82^T^ showing synteny of genes among the genome

We used MAUVE [49] for whole genome alignment of 10.1601/nm.17912 strain USBA 82^T^ with 10.1601/nm.4488 type strain (10.1601/strainfinder?urlappend=%3Fid%3DSEBR+4207 ^T^). The alignment showed conserved clusters and synteny of the majority of the genes (Fig. 3). However, there are some rearrangements dispersed in the genome of 10.1601/nm.17912. There is a clear inversion of two regions at the end of the genome and small translocations of regions. Those differences are consistent with the phylogenetic distance between the two species.

Metabolic information contained in the genome of 10.1601/nm.17912 includes genes related to amino acid transport and metabolism, thiosulfate reduction, and heat shock proteins (hsps). Ammonification genes, mainly nitrate reductase genes (*narG,H,I,J*), were also observed throughout the genome. In addition, the presence of proline operon *proHJ* and p*roA* gene could be related to the response to high osmolarity through de novo synthesis of proline to protect the cell from stress [50].

The fermentation of amino acids observed in this species is more commonly found in the phylum 10.1601/nm.14317, which have a high proportion of amino acid transport and metabolism genes (COG E), than in any other bacterial phylum to date [48]. 10.1601/nm.17912 contained a total of 229 genes related to this COG category. This represents 11.8% of the genes of this genome.

In contrast, carbohydrate fermentation has only been exhibited by a few cultured species in the phylum 10.1601/nm.14317, such as 10.1601/nm.4504 [51] and 10.1601/nm.7999 spp. [14, 52, 53]. These observations, based on cultured members of the phylum 10.1601/nm.14317 *,* suggest that members of this phylum are specialists with relatively shallow ecophysiological niches [3]. As was expected, only 5.5% of the genes in the genome of 10.1601/nm.17912 were categorized as carbohydrate transport and metabolism genes.

IMG tools were used to identify nine biosynthetic gene clusters that are associated with secondary metabolites. With the exception of a cluster reported as a bacteriocin, clusters were identified as putative. antiSMASH 3.0.5 was used to detect 11 clusters of biosynthetic genes related to bacteriocins (18.2%), fatty acids (18.2%), lipopolysaccharide (9.1%) and putative biosynthetic clusters (11%). We found that one of the putative biosynthetic clusters is related to exopolysaccharide (EPS) production. This cluster includes an EPS biosynthesis domain protein, a polysaccharide export protein, a sugar transferase, a nucleotide sugar dehydrogenase and a NAD-dependent epimerase/dehydratase. It has been reported that EPS of benthic bacteria is involved in motility [54], in absorbing nutrient elements [55] and in assisting attachment of bacteria to organic particles and other surfaces [56]. The presence of this biosynthetic cluster related to EPS production could be an adaptive advantage for growth of this strain in its natural habitat. Using BAGEL3, we identified two biosynthetic clusters. The bacteriocin Linocin M18-like structural protein (>10KDa) (BAGEL3 bacteriocin III database PF04454.7 [1.8e-80] - BlastP 3e-143) belongs to the peptidase U56 family (see Additional file 9: Figure S9a). It presents a similarity of 73% with the Linocin-M18 protein identified in 10.1601/nm.4488. The other cluster was a sactipeptide (see Additional file 9: Figure S9b), but there were no significant BlastP hits for the putative structural gene product. We also identified a gene related to a transposase (BlastP 2e-33) in this cluster. This gene is frequently found in association with bacteriocins, but we also found a putative ABG transporter (PF03806.8 [5.8e-148] – BlastP 0.0) and genes predicted to encode a radical SAM (S-adenosylmethionine) which are involved in bacteriocin maturation (PF14319.1 [9.2e-05] - BlastP 3e-147).

## Conclusions

The genome of 10.1601/nm.17912 USBA 82^T^ provides insights into many aspects of its physiology and evolution. Sequence analysis and comparative genomics corroborated the taxonomic affiliation of 10.1601/nm.17912 into the 10.1601/nm.14317 phylum. We detected six of the seven conserved signature indels (CSIs) identified by Bhandari and Gupta [24] as useful for distinguishing the species of the phylum. Our results grouped 10.1601/nm.11530 *,* 10.1601/nm.14314 and 10.1601/nm.4487 species together and confirmed the specificity of these CSIs in highly conserved regions of proteins as targets for evolutionary studies in 10.1601/nm.14317.

The genome of 10.1601/nm.17912 USBA 82^T^ contains genes related to amino acid transport and metabolism, thiosulfate reduction and ammonification. This agrees with experimental data and physiological observations. The presence of proline operon genes demonstrates the possibility of a cellular response to high osmolarity through de novo synthesis of proline to protect the cell from stress. Using our bioinformatics workflow, we identified bacteriocin genes associated with secondary metabolites in the genome. Future research will address whether or not these clusters of biosynthetic genes express the associated secondary metabolites that we have identified.

## Additional files

Additional file 1: Figure S1. Phylogenetic relationships of *D. salsuginis* USBA 82^T^ based on analysis of 16S rRNA gene sequencing. The evolutionary history was inferred by using the Maximum Likelihood method based on the General Time Reversible model. The percentage of trees in which the associated taxa clustered together is shown next to the branches. Initial tree(s) for the heuristic search were obtained automatically by applying Neighbor-Joining and BioNJ algorithms to a matrix of pairwise distances estimated using the Maximum Composite Likelihood (MCL) approach, and then selecting the topology with superior log likelihood value. A discrete Gamma distribution was used to model evolutionary rate differences among sites (5 categories (+G, parameter = 0.6072)). The rate variation model allowed for some sites to be evolutionarily invariable ([+I], 46.4848% sites). The tree is drawn to scale, with branch lengths measured in the number of substitutions per site. The analysis involved 26 nucleotide sequences. All positions containing gaps and missing data were eliminated. There was a total of 1090 positions in the final dataset. Evolutionary analyses were conducted in MEGA 7. (DOCX 72 kb)

Additional file 2: Figure S2. Multiple alignment of Penicillin binding protein, 1A family. Multiple alignment contained a 4 aa deletion which is specific for *Dethiosulfovibrio, Jonquetella and Pyramidobacter* clade. The analysis was done using Mafft. (DOCX 34 kb)

Additional file 3: Figure S3. Multiple alignment of the adenosylcobalamin-dependent ribonucleoside-diphosphate reductase protein. The multiple alignment contained a 1 aa insertion which is specific for *Dethiosulfovibrio, Jonquetella and Pyramidobacter* clade. The analysis was done using Mafft. (DOCX 34 kb)

Additional file 4: Figure S4. Multiple alignment of a conserved region of the DNA directed RNA polymerase, ß subunit (RpoB) protein. The multiple alignment contained a 13 aa insertion that is specific for *Dethiosulfovibrio, Jonquetella and Pyramidobacter* clade. The analysis was done using Mafft. (DOCX 34 kb)

Additional file 5: Figure S5. Multiple alignment of a conserved region of the 1-acyl-sn-glycerol-3- phosphate acyltransferase protein. The multiple alignment contained a 1 aa insertion that is specific for *Dethiosulfovibrio, Jonquetella and Pyramidobacter* clade. The analysis was done using Mafft. (DOCX 36 kb)

Additional file 6: Figure S6. Multiple alignment of a conserved region of the e tRNA modification GTPase TrmE protein. The multiple alignment contained a 1 aa insertion that is specific for *Dethiosulfovibrio, Jonquetella and Pyramidobacter* clade. The analysis was done using Mafft. (DOCX 34 kb)

Additional file 7: Figure S7. Multiple alignment of the Putative DEAD/DEAH box helicase proteins. CSIs previously reported in this protein were not found. The analysis was done using Mafft. (DOCX 46 kb)

Additional file 8: Figure S8. Partial sequence alignment of the Hsp60 protein. The sequence alignment is showing the absence of 1 aa (red) in a conserved region that is mainly specific for atypical diderm taxa (*Negativicutes*, ‘*Fusobacteria’*, *Synergistetes* and ‘*Elusimicrobia’*) from all of the phyla of traditional Gram-negative bacteria that contain this insert. Only representative sequences from different bacterial phyla are shown here. Accession numbers of the non-redundant protein database are: *Escherichia coli* WP_077064857.1, *Nostoc commune* BAF95909.1, *Helicobacter pylori* WP_020981906.1, *Lentisphaera araneosa* WP_007279303.1, *Rickettsia prowazekii* WP_004596265.1, *Pseudomonas aeruginosa* WP_050442419.1, *Ralstonia solanacearum* WP_013213354.1*, Bacillus subtilis* WP_087960787.1, *Aminomonas paucivorans* WP_006301345.1, *Dethiosulfovibrio salsuginis* WP_085544335.1. (DOCX 26 kb)

Additional file 9: Figure S9. Diagrammatic representation of A) the Linocin-M18 like gene clusters and B) Sactipeptides like gene clusters. (DOCX 64 kb)

## Abbreviations

CMPUJU: Colección de Microorganismos de la Pontificia Universidad Javeriana
CSIs: Conserved signature indels
DSM: Deutsche Sammlung von Mikroorganismen
IDA: inferred from direct assay
Indels: insertions/deletions
KCTC: Korean Collection for Type Culture
m.a.s.l: meters above sea level
MIGS: Minimum information about a genome sequence
NAS: Non-traceable author statement
TAS: Traceable author statement
WDCM: World Data Center for Microorganisms
