# Supplementary material for: Draft genome sequence of Dethiosulfovibrio salsuginis DSM 21565T an anaerobic, slightly halophilic bacterium isolated from a Colombian saline spring
Source: Stand Genomic Sci. 2017 Dec 20;12:86. doi: 10.1186/s40793-017-0303-x (PMC5738826; doi:10.1186/s40793-017-0303-x)
Supplement: Supplementary file 1 — Phylogenetic relationships of D. salsuginis USBA 82T based on analysis of 16S rRNA gene sequencing. The evolutionary history was inferred by using the Maximum Likelihood method based on the General Time Reversible model. The percentage of trees in which the associated taxa clustered together is shown next to the branches. Initial tree(s) for the heuristic search were obtained automatically by applying Neighbor-Joining and BioNJ algorithms to a matrix of pairwise distances estimated using the Maximum Composite Likelihood (MCL) approach, and then selecting the topology with superior log likelihood value. A discrete Gamma distribution was used to model evolutionary rate differences among sites (5 categories (+G, parameter = 0.6072)). The rate variation model allowed for some sites to be evolutionarily invariable ([+I], 46.4848% sites). The tree is drawn to scale, with branch lengths measured in the number of substitutions per site. The analysis involved 26 nucleotide sequences. All positions containing gaps and missing data were eliminated. There was a total of 1090 positions in the final dataset. Evolutionary analyses were conducted in MEGA 7. (DOCX 72 kb) [file 40793_2017_303_MOESM1_ESM.docx]

*Dethiosulfovibrio marinus* DSM 12537^T^ (AF234544)

*Dethiosulfovibrio acidaminovorans* DSM 12590^T^ (AY005466)

*Dethiosulfovibrio russensis* DSM 12538^T^ (AF234542)

*Dethiosulfovibrio peptidovorans* DSM 11002^T^ (ABTR01000010)

***Dethiosulfovibrio salsuginis* USBA 82^T^=DSM 21565^T^ (EU719657**)

*Jonquetella anthropi* DSM 22815^T^ (CM001376)

*Pyramidobacter piscolens* DSM 21147^T^(EU309492)

*Rarimicrobium hominis* CCUG 65426^T^ (EF468685)

*Aminobacterium thunnarium* DSM 27500^T^ (KJ159211)

*Aminobacterium colombiense* DSM 12261^T^ (CP001997)

*Aminobacterium mobile* DSM 12262^T^ (JAFZ01000002)

*Aminivibrio pyruvatiphilus* DSM 25964^T^ (AB623229)

*Lactivibrio alcoholicus* DSM 24196^T^ (AB558582)

*Fretibacterium fastidiosum* DSM 25557^T^ (GQ149247)

*Aminiphilus circumscriptus* DSM 16581^T^ (AY642589)

*Thermanaerovibrio velox* DSM 12556^T^ (CM001377)

*Aminomonas paucivorans* DSM 12260^T^ (AF072581)

*Synergistes jonesii* ATCC 49833^T^ (L08066)

*Cloacibacillus evryensis* DSM 19522^T^ (CU463952)

*Cloacibacillus porcorum* DSM 25858^T^ (JQ809697)

*Thermovirga lienii* DSM 17291^T^ (CP003096)

*Acetomicrobium flavidum* DSM20664^T^ (FR733692)

*Anaerobaculum* mobile DSM 13181^T^ (AJ243189)

*Anaerobaculum hydrogeniformans* ATCC BAA-1850^T^ (FJ862996)

*Anaerobaculum thermoterrenum* DSM 13490^T^ (U50711)

*Thermodesulfobacterium indicus* DSM 15286^T^ (AF393376)

100

99

100

100

100

100

94

100

100

100

83

100

97

69

98

73

99

63

52

82

86

0.05

**Additional file 1:** **Figure S1.docx**.
