# Supplementary figures and images for: Draft genome sequence of Dethiosulfovibrio salsuginis DSM 21565T an anaerobic, slightly halophilic bacterium isolated from a Colombian saline spring
Source: Stand Genomic Sci. 2017 Dec 20;12:86. doi: 10.1186/s40793-017-0303-x (PMC5738826; doi:10.1186/s40793-017-0303-x)

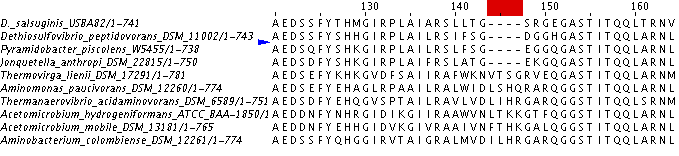


**Additional file 2: Figure S2.**

Supplement: Supplementary file 2 — Multiple alignment of Penicillin binding protein, 1A family. Multiple alignment contained a 4 aa deletion which is specific for Dethiosulfovibrio, Jonquetella and Pyramidobacter clade. The analysis was done using Mafft. (DOCX 34 kb) [file 40793_2017_303_MOESM2_ESM.docx]

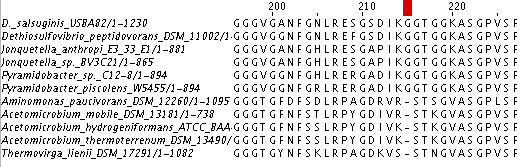


**Additional file 3:** **Figure S3.**

Supplement: Supplementary file 3 — Multiple alignment of the adenosylcobalamin-dependent ribonucleoside-diphosphate reductase protein. The multiple alignment contained a 1 aa insertion which is specific for Dethiosulfovibrio, Jonquetella and Pyramidobacter clade. The analysis was done using Mafft. (DOCX 34 kb) [file 40793_2017_303_MOESM3_ESM.docx]

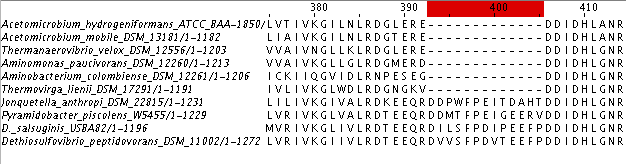


**Additional file 4:** **Figure S4.**

Supplement: Supplementary file 4 — Multiple alignment of a conserved region of the DNA directed RNA polymerase, ß subunit (RpoB) protein. The multiple alignment contained a 13 aa insertion that is specific for Dethiosulfovibrio, Jonquetella and Pyramidobacter clade. The analysis was done using Mafft. (DOCX 34 kb) [file 40793_2017_303_MOESM4_ESM.docx]

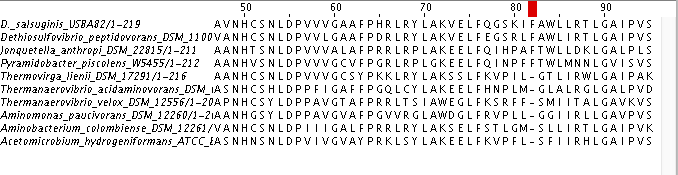


**Additional file 5:** **Figure S5**.

Supplement: Supplementary file 5 — Multiple alignment of a conserved region of the 1-acyl-sn-glycerol-3- phosphate acyltransferase protein. The multiple alignment contained a 1 aa insertion that is specific for Dethiosulfovibrio, Jonquetella and Pyramidobacter clade. The analysis was done using Mafft. (DOCX 36 kb) [file 40793_2017_303_MOESM5_ESM.docx]

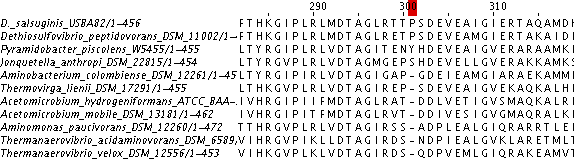


**Additional file 6:** **Figure S6.**

Supplement: Supplementary file 6 — Multiple alignment of a conserved region of the e tRNA modification GTPase TrmE protein. The multiple alignment contained a 1 aa insertion that is specific for Dethiosulfovibrio, Jonquetella and Pyramidobacter clade. The analysis was done using Mafft. (DOCX 34 kb) [file 40793_2017_303_MOESM6_ESM.docx]

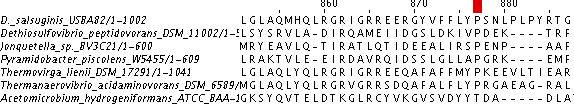


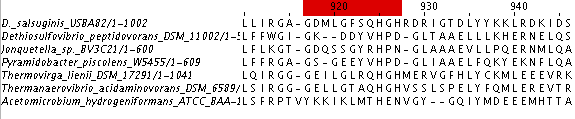


**Additional file 7: Figure S7.**

Supplement: Supplementary file 7 — Multiple alignment of the Putative DEAD/DEAH box helicase proteins. CSIs previously reported in this protein were not found. The analysis was done using Mafft. (DOCX 46 kb) [file 40793_2017_303_MOESM7_ESM.docx]

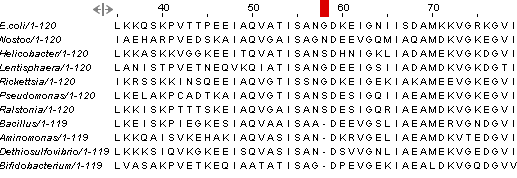


**Additional file 8:** **Figure S8.**

Supplement: Supplementary file 8 — Partial sequence alignment of the Hsp60 protein. The sequence alignment is showing the absence of 1 aa (red) in a conserved region that is mainly specific for atypical diderm taxa (Negativicutes, ‘Fusobacteria’, Synergistetes and ‘Elusimicrobia’) from all of the phyla of traditional Gram-negative bacteria that contain this insert. Only representative sequences from different bacterial phyla are shown here. Accession numbers of the non-redundant protein database are: Escherichia coli WP_077064857.1, Nostoc commune BAF95909.1, Helicobacter pylori WP_020981906.1, Lentisphaera araneosa WP_007279303.1, Rickettsia prowazekii WP_004596265.1, Pseudomonas aeruginosa WP_050442419.1, Ralstonia solanacearum WP_013213354.1, Bacillus subtilis WP_087960787.1, Aminomonas paucivorans WP_006301345.1, Dethiosulfovibrio salsuginis WP_085544335.1. (DOCX 26 kb) [file 40793_2017_303_MOESM8_ESM.docx]

**A**


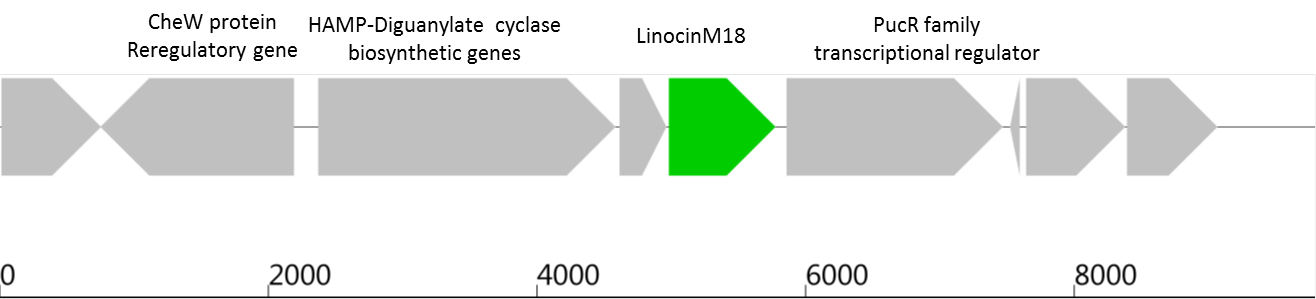


**B**


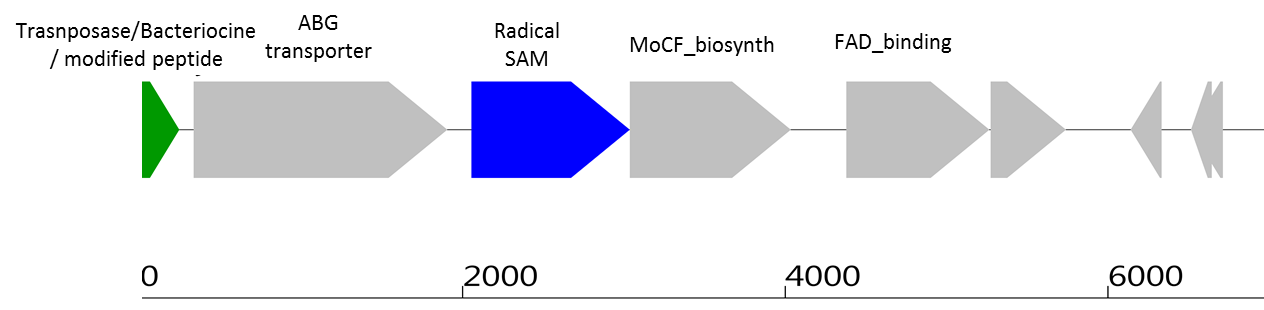


**Additional file 9: Figure S9**.

Supplement: Supplementary file 9 — Diagrammatic representation of A) the Linocin-M18 like gene clusters and B) Sactipeptides like gene clusters. (DOCX 64 kb) [file 40793_2017_303_MOESM9_ESM.docx]
